# Notes and News

**Published:** 1895-04-20

**Authors:** 


### Notes and News.

(Collected and Communicated.)

The eighth Universal Cookery and Food Exhibition will be opened at the Portman Rooms by Prince Edward of Saxe-Weimer, K.C.B., on May 7th. Many of the County Council Technical Education Committees are intending to send pupils to compete for the Cookery Scholarships.

Mr. J. Pierpoint Morgan has just given a donation of 20,000 dollars towards the building of a Sanitarium for Consumptive Patients at Liberty, Sullivan County. The site has been already purchased, and consists of two hundred acres of land.

Sir Joseph Lister was presented, on April 9th, by the Prince of Wales, with the Albert Medal of the Society of Arts in recognition of his great services to the cause of modern surgery by "the discovery and establishment of the antiseptic method of treating wounds and injuries, by which not only has the art of surgery been equally promoted and human life saved in all parts of the world, but extensive industries have also been created for the supply of materials required for carrying the treatment into effect." Many members of the Council of the Society of Arts, of which the Prince of Wales is president, were present at Marlborough House on the occasion.

A committee has been formed to carry out the plan for the endowment of a cot in the Great Ormond Street Children's Hospital in memory of Corney Grain, upon which the Duke of Beaufort, the Earls of Wharncliffe and Onslow, the Marquis of Granby, Sir Algernon Borthwick, Mr. Comyns Carr, Mr. George Grossmith, Mr. John Hare, amongst others, have consented to serve. Subscriptions may be sent to Messrs. Drummond, 49, Charing Cross, or to the Honorary Treasurers, Mr. R. C. Lehmann, 30, Bury Street, St. James's, and Mr. T. D. Croft, Grafton Galleries, Grafton Street. The committee wish the fund to be thoroughly representative, and will therefore gratefully receive any subscriptions, however small.

The Duchess of Teck has consented to visit the Chelsea Hospital for Women early in May, when her Royal Highness will open and name a ward and receive purses of money from those who are willing to aid the funds of this institution.

On Wednesday last the Duchess of Teck opened a bazaar at Derby on behalf of the funds of the Derbyshire Royal Infirmary.—On the same day Princess Christian was present at a similar function at Iver, Bucks, in aid of the local Cottage Hospital.—Her Royal Highness the Duchess of Albany, accompanied by Miss Heron Maxwell, visited St. Saviour's Hospital, Osnaburgh Street, last week.

A drawing-room meeting was recently held at the residence of Sir Thomas Fowell Buxton on behalf of the Sisters and Nurses' Branch of the Trinity College, Cambridge, Mission. H.R.H. the Duchess of York was present, and a number of ladies interested in the work. An appeal was made by the Rev. R. Appleton for funds to increase the number of workers, only five ladies being at present employed. The Bishop of Southwark, Canon Fleming, and the Master of Trinity were amongst the speakers.

The people of Walsall are making energetic efforts to raise funds for the repair of their hospital, after the disaster wrought by the late gale. Indeed, so successful have already been the results of these efforts, that it is hoped a sufficiently substantial sum will be collected to enable rebuilding on more modern lines to be carried out, and also the provision of a medical department. If these improvements are accomplished, the recent storm will have proved itself a blessing in disguise.

The report just presented by the medical officer of the Local Government Board regarding the cholera in England in 1893 details many facts which appear to have an important bearing on the question which has recently so agitated the public mind as to the possibility of oysters and other molluscs becoming a medium of infection. It seems to be quite clear that in several cases in which cholera arose in inland towns Grimsby or Cleethorpes was the starting point of the infection, and that the specific cause of the illness was in some instances the consumption of oysters or other shellfish, either eaten at the place or brought from it. The conditions under which the culture of these molluscs is carried out would certainly seem to favour their infection, if such infection be possible. The foreshore opposite Grimsby and Cleethorpes is very shallow, and when the tide goes down it leaves a great expanse of mud or sand flats, which are famous as oyster, mussel, and cockle beds. The sewage from both these places pours out over these flats at low water and is swept back over them by the returning tide, and in view of the fact that there undoubtedly was cholera in Grimsby, and that cholera arose in persons in inland towns who were known to have consumed these shellfish, we can hardly avoid agreeing with Dr. Thorne Thorne, who says: "Having regard to the significant indications afforded by some of the cholera histories of last year, I cannot avoid the conviction that shell fish from Cleethorpes and Grimsby must, in some cases, remain under suspicion as having contributed to the diffusion of the disease." This is important in regard to the question so recently discussed. *Ex uno disce omnes.* If cholera can be so spread, why not typhoid?

